# Causes of elective surgery cancellation and theatre throughput efficiency in an Australian urology unit

**DOI:** 10.12688/f1000research.4824.1

**Published:** 2014-08-19

**Authors:** Andrew Keller, Akbar Ashrafi, Ahmad Ali

**Affiliations:** 1 Department of Urology, Ipswich General Hospital, Chelmsford Ave, Ipswich QLD, 4305, Australia

## Abstract

**Objective:**

- To evaluate our unit’s theatre throughput efficiency, to identify where inefficiencies existed and consequently where the greatest improvement might be made.
- To identify the causes of day of surgery cancellations and how they might be avoided. 

**Patients and Methods:**

- A prospective audit of theatre utilisation was undertaken over a 6 month period between 05/02//2013 and 02/08/2013 at Ipswich General Hospital, QLD, Australia.
- Times collected were: time of patient arrival in anaesthetic bay, start time of operative procedure, end time of operative procedure, and time of patient leaving theatre.
- The causative factors for any delays or day of surgery cancellations were identified and recorded where possible.

 
**Results:**

- In the six month period 26,850 sessional minutes were available for elective operating over 100 operating sessions.
- 304 elective cases were performed, split between 21 major and 283 minor procedures
- The sessions ran overtime a cumulative 2114 minutes.
- Total non-operative minutes totalled 13,209 (50.3% of all available time), split between late starts 499 minutes (1.8%), early list finishes 1894 minutes (7.05%),  changeover time 1869 minutes (6.9%) and anaesthetic time, 8974 minutes (33.4%)
- Actual operating time only compromised 50.7% of all available elective operating session time (13,614 minutes)
- Theatre utilisation was 91.8%.
- 51 procedures were cancelled on the day of surgery during the audit period, representing 14.3% of all scheduled procedures.
- The most common reason for cancellation was lack of surgical fitness, followed by inadequate operative time.

 
**Conclusion:**

-  A significant proportion of all elective operative time was consumed by non-operative minutes.
- Inefficiencies existed in turnover of patients as well as over as well as underbooking of patients on elective lists.
- An excessive number of cases were cancelled on the day of surgery, wasting valuable operative time.
- A multi-parametric approach must be taken to improve operation list utilisation.

## Introduction

Theatre efficiency is increasingly coming under the spotlight as elective waiting lists continue to increase ^1^. Delays or interruptions during operating lists are associated with dissatisfaction for health care providers and patients alike ^2^. Theatre lists account for a significant proportion of a hospital’s revenue and an even larger fraction of its total expenses ^3– 6^. As operating theatre budgets are already stretched, increased case throughput must come from improved theatre efficiency rather than from more operating sessions.

Efficient use of theatre sessions relies on prompt start times, an appropriately booked theatre case-mix, efficient patient turnover, and finishing on time to reduce overtime costings ^7, 8^. Accurate scheduling of elective theatre cases to maximise operating efficiency is extremely complex, as the time required for identical procedures can vary dramatically.

The most cost-efficient method to increase theatre case throughput is by decreasing idle theatre time ^9, 10^.

## Method

We sought to evaluate our unit’s theatre throughput efficiency so we might identify areas where the most time was wasted during operating sessions, and consequently where the most significant improvements might be made.

To do this we undertook a prospective audit of all elective theatre operating in the Urology unit at Ipswich General Hospital (IGH), a regional secondary referral hospital, over a six month period, between 05/02//2013 and 02/08/2013. The theatre complex at IGH consists of 6 operating suites with a 3 bay arrangement, with each suite having an anaesthetic bay and scrub room in addition to the operating room itself.

Times were extracted from the Operating Room Management Information System (ORMIS) theatre management software (CSC). Versions 5 and subsequently 7 were used, as the software was updated during the audit period. Times extracted from ORMIS were: time of arrival in anaesthetic bay, start time of operative procedure, end time of operative procedure, and time of patient leaving theatre ( Table 1). Times were entered into ORMIS by theatre nursing staff as per standard practice.

**Table 1.** Times extracted from Operating Room Management Information System (ORMIS) theatre management software (CSC) during the audit period, listing the various steps during a patient’s operative journey.

Times Recorded	Abbreviation
Time of patients arrival in anaesthetic bay	T1
Start time of operation	T2
End time of operation	T3
Time of patient departure from theatre	T4

Where possible the reasons for delays were identified and recorded by both the nursing staff entering the reasons into ORMIS, and by direct recording by a surgical team observer.

The scrub nurses, theatre assistants, and members of the anaesthetic team were not informed that the study was being conducted, so as not to influence their performance. The surgical team were undertaking the audit, and they were never blinded.

## Results

In the six month audit period 304 elective cases were performed, split between 21 major ( Table 2) and 283 minor procedures ( Table 3). Total available operative minutes were 26,850 distributed over 100 elective operating lists. Ordinarily each week consisted of one 3.5 hour, one 8.5 hour and two 3 hour sessions.

**Table 2.** Breakdown of the major operative procedure performed during the audit period.

Major Operations	No. Performed
Lap/Open Nephrectomy	6
Lap/Open Nephro-ureterectomy	1
Lap/Open Pyeloplasty	1
Percutaneous Nephrolithotomy (PCNL)	2
Ureteric Reimplantation	1
Artifical Urinary Sphincter	1
E/O skin lesion + skin graft	1
Radical Retropubic Prostatectomy (RRP)	8
Total	21

**Table 3.** Breakdown of minor operative procedure case-mix during the audit period.

Minor Operations	No.	Minor Operations	No.
Ureteroscopy	58	Removal of ureteric stent	14
Conduitoscopy	1	Cystoscopy	25
Inguinal Orchidectomy	8	Cystoscopy + Retrograde Pyelogram	3
Cystoscopy + biopsy	19	Cystoscopy + urethral dilatation	5
Scrotal lesion excision/ abscess drainage	4	Incision of ureterocoele	2
Continence sling	5	Cystoscopy + injection of intravesical botox	2
I/O Ureteric stent	29	Hydrocoele repair	2
Trans-Urethral Resection of Bladder Tumour (TURBT)	35	TRUS biopsy under sedation	7
Bladder Neck Incision (BNI)	4	Trans-Urethral Resection Prostate (TURP)	34
I/O Suprapubic Catheter (SPC)	1	Cystoscopy + diathermy	4
Circumcision	9	Optical urethrotomy	5
Open Cystolithotomy	1	E/O Epididymal Cyst	6
Total			283

Start time efficiency or late starts (LS) ( Table 4), measured from the time the patient entered the anaesthetic bay, was acceptable at 499 minutes or 1.8% of all available list time ( Table 5). Significant LS (over 15 minutes late) occurred on only 8 lists. Over all operating lists the mean LS was 5 minutes with a median of 0 minutes, and a range of 0–148 minutes. The total LS time was skewed significantly by a single episode where 6 nursing staff were absent with illness simultaneously, delaying the start of the list by 148 minutes. Only a small number of cases were delayed by the late arrival of anaesthetic or surgical team members. Delay in patients arriving from the day surgery unit or wards were more common but still infrequent.

**Table 4.** Definitions of times extrapolated from the ORMIS data recorded during the audit period.

Definition of Times	Explanation of Times
Sessional time (ST)	All time within the allocated elective operating session excluding OT + ES time
Early start time (ES)	Scheduled start of ST – T1
Late start time (LS)	T1 - scheduled start time of ST
Anaesthetic Time (AT)	(T2 – T1) + (T4 – T3)
Changeover Time (CT)	T1 (next patient) – T4 (previous patient)
Procedure Time (PT)	T3 – T2
Overtime (OT)	PT + AT occurring after end of ST
Non-operative Time (NOT)	AT + CT

**Table 5.** Breakdown of ST usage. Just over 50% of all available sessional time was used for operating during the audit period.

Activity	Total Minutes	Mean (Overall)	Median (Overall)	Range (Overall)	No. Significant	Mean (Significant)	Median (Significant)	Range (Significant)	% ST
LS	499	5	0	0–148	8	53.38	39.50	25–148	1.80
EF	1894	N/A	N/A	N/A	24	76.25	45	18–480	7.05
CT	1869	8.16	5	0–132	31	31.61	14	16–132	6.96
AT	8974	29.52	21.50	2–64	N/A	N/A	N/A	N/A	33.42
PT	13614	44.78	31	2–330	N/A	N/A	N/A	N/A	50.70

1894 minutes were wasted with early finishing (EF) ( Table 4) of lists representing 7.05% of all available time ( Table 5). Significant EF, considered as lists finishing over 15 minutes early, affected 24 lists and totalled 1830 minutes, with a mean of 76.25 minutes, a median of 45 and a range of 18–480 minutes.

Under booking of theatre lists accounted for a significant proportion of all EF, however, the lion’s share of early list finishes were caused by day of surgery cancellations. 27% of all EF (843 minutes) were accounted for by cancellation of just 3 cases ( Table 6). Two radical retro-pubic prostatectomies (RRP) were cancelled due to patients changing their mind on the day of surgery and instead opting for external beam radiation therapy, with a cumulative loss of 363 minutes of scheduled sessional time (ST) ( Table 4). The cancellation of a radical cystectomy, which was the only booked case on an all-day operating list, accounted for 480 lost minutes.

**Table 6.** Breakdown of cases cancelled on the day of surgery and the reasons for their cancellation. Cancellation reasons are classified as per Argo *et al’s* cancellation codes ^11^.

Cancelled Operations	No.	Reason For Cancellation	No.
**MAJOR**			
RRP	1	W5	2
Radical Cystectomy + Ileal Conduit Formation	1	W1	1
**MINOR**			
TURBT	8	W4	4
		W3(UTI)	2
		F6	1
		P1 (Trauma witnessed)	1
TURP	7	W4	2
		W3(UTI)	1
		F7	2
		F11	1
		A1	1
Cystoscopy + I/O Ureteric stent	6	P8	1
		W4	2
		W3(UTI)	2
		P10	1
Optical Urethrotomy	1	F7	1
Cystoscopy	6	W4	4
		W3(UTI)	1
		W5	1
Cystoscopy _ RGP	2	F6	1
		F7	1
Ureteroscopy	4	F7	2
		W3(UTI)	1
		W4	3
		W2	1
Hydrocoele Repair	2	P10	1
		F7	1
Circumcision	3	W4	2
		F7	1
Cystoscopy +/- biopsy	6	W4	2
		P6	1
		F7	2
		W2	1

In total, 51 procedures were cancelled on the day of surgery during the audit period, representing 14.3% of all scheduled procedures. The reasons for case cancellation were grouped into 5 categories, and 28 potential cancellation reasons, as per Argo *et al’s* audit of elective operating in the US Veteran’s Health Administration ^11^ ( Table 7). The most common reason for case cancellation was lack of fitness for surgery (W4), with inadequate operative time the second most common (M7) ( Table 8).

**Table 7.** Classification of cancellation codes used to group day of surgery (DOS) cancellations. **Source:** Adapted from: Argo JL, Vick CC, Graham LA, Itani KM, Bishop MJ, Hawn MT. Elective surgical case cancellation in the Veterans Health Administration system: identifying areas for improvement. *Am J Surg* 2009;198:600–6.

Patient	Facility
P1 Patient refused or no consent	F1 Equipment broken or not available
P2 VA transportation	F2 Implant(s) not available
P3 Patient transportation	F3 No Intensive Care Unit (ICU) beds
P4 Preoperative instructions not followed or patient not instructed adequately	F4 No Hospital beds
P5 Patient substance	F5 Scheduling error
P6 Patient cancels, had procedure performed elsewhere	F6 Staff shortage, other than surgeons and anaesthesia providers
P7 Patient cancels, did not have procedure performed elsewhere	F7 No OR time
P8 Patient death	F8 Emergency case
P9 Case aborted in OR	F9 Blood products not available
P10 Patient is a no-show, no contact from patient	F10 Facility environment
**Work-up**	F11 Weather/natural disaster
W1 Surgeon-work up needed	**Anaesthesia**
W2 Anaesthesia-work up needed	A1 Anaesthesia staff not available
W3 Abnormal test	**Surgeon**
W4 Change in medical status	S1 Surgery staff not available
W5 Change in treatment plan	

**Table 8.** The frequency of individual cancellation classification codes used for day of surgery cancellations during the audit period. Cancellation reasons are classified as per Argo *et al’s* cancellation codes ^11^.

Reason for Cancellation	Number of Cancellations
Patient refuses operation	2 (P1)
Procedure already performed elsewhere	1 (P6)
Patient deceased	1 (P8)
Failed to attend	2 (P10)
Patient inadequately prepared for surgery (Not bowel prepped)	1 (W1)
Inadequate anaesthetic workup	2 (W2)
UTI on dipstick (Clinically well)	6 (W3)
Patient generally unwell	17 (W4A)
Patient no longer needs procedure	3 (W4B)
Patient no longer wants procedure	3 (W5)
List reduced due to staff training	2 (F6)
Lack of operative time	10 (F7)
Lack of anaesthetic staff	1 (A1)
**Total**	**51**

2114 minutes were recorded of theatre overtime (OT) ( Table 4), measured from the time patients left the operating room. This represented 7.9% overtime over the scheduled ST during the audit period ( Table 5). Operative OT, measured from completion of the last operative procedure accounted for 1404 minutes (66.4%), with anaesthetic overtime accounting for the remaining 710 minutes (33.6%). Significant OT affected 37 operating lists, with a mean of 54.59 and median of 37 minutes and a range of 16–105 minutes. The causes of OT during the audit were multifactorial. Any unforeseen delays during the operative list, such as a slow patient changeover, difficult induction of anaesthesia, late start of the operative list, or unexpectedly prolonged operative time all contributed to total overtime.

One of the major contributors that was identified was major cases being booked onto half day operating lists. Two lists each week were of only 180 minutes duration, and on 4 out of 5 occasions when a major case was booked onto such a list, the session ran significantly overtime.

Total patient changeover time (CT) ( Table 4), which was defined as the time the patient left the operating room until the subsequent patient on the operating list entered the anaesthetic bay was acceptable at 1869 minutes, representing 6.9% of all available operative list time ( Table 5). The mean CT was 8.16 minutes, the median 5 minutes with a range of 0–132 minutes. A significant delay in CT, defined as those taking over 15 minutes, occurred on 31 occasions (13.4% of all changeovers). Late patient arrival or non-arrival at the day of surgery admissions was responsible for a significant proportion of all CT. The next patient was already in the anaesthetic bay before completion of the prior case on 60 occasions (26% of all changeovers), significantly reducing total CT.

Anaesthetic time (AT) ( Table 4) which consisted of: patient time spent in the anaesthetic bay, anaesthetic induction time and the time for the patient to leave theatre after the end of the procedure, totalled 9657 minutes. After removing the AT spent in overtime (710 minutes), AT consumed 33.4% of all available operating list time. Mean anaesthetic time over the audit was 29.52 minutes, with a median of 21.5 and a range of 2–64 minutes.

Of all available time, 15018 minutes were spent operating (PT) ( Table 4). After excluding the PT occurring after the scheduled end of the operating list (1404 minutes), this meant that only 50.7% of all available sessional time (ST) was spent operating ( Table 5).

Theatre utilisation over the entire audit period was 91.8%, however, this number was significantly skewed by the large amount of both OT and early start minutes (ES). ES, measured from entry of the patient into the anaesthetic bay prior to the scheduled start of the operating list, totalled 967 minutes. When these minutes, in addition to the OT (2114 minutes), are subtracted effective theatre utilisation falls to 80.3%.

There were several limitations to our study. While we attempted to blind the theatre assistants, anaesthetic and nursing staff from the ongoing audit, several members of each team became aware of the audit throughout its course. This could have influenced their efforts throughout the audit period. As all times for the study extracted from ORMIS were entered by nursing staff, it is possible that bias could have affected the accuracy of the times if the nurses entering the data were aware of the audit. The surgical team was never blinded to the audit, and this might have influenced the operative urgency of the surgeons involved and their punctuality.

In regards to our case mix, our relatively small proportion of major cases compared to a tertiary referral urology service would certainly increase the ratio of non-operative to operative time compared to an operative case mix with more major cases.

Another factor that influenced our throughput was addition of emergency cases to our elective lists. Ipswich General Hospital has one emergency list daily, which preferentially performs all emergency cases unless elective sessions finish early, or if the patient is medically unstable. During the audit period 4 emergency cases were added to the end of our elective list: 1 drainage of a scrotal abscess and 3 ureteric stents. Total overtime generated by these additional cases totalled 106 minutes. If these additional cases had not been performed, an additional 114 early finishing minutes would have been recorded.

Raw data files (Excel) for the audit of elective theatre operating in the Urology unit, Ipswich General Hospital, QLD, Australia File 1: Elective Operating Session Time Usage.xlsx, Elective operating session time usage as obtained from the ORMIS theatre management software package. File 2: Elective Surgery Cancellation Causes.xlsx, reasons for cancellation of surgery and the ORMIS code allocated to each cancelled procedure. DOS, day of surgery.

## Discussion

Our audit has highlighted the complexity of maximising operative efficiency. Optimisation of theatre throughput efficiency starts with careful booking of the operating list. Currently operating lists are booked by ex-clinical staff with a best-guess approach, which while practical, often fails to take into account the myriad variables of the case and staffing mix. In various centres mathematical theories previously applied to the manufacturing industry have been successfully trialled to facilitate more efficient booking of theatre time, however, such methods require significant expertise and staff retraining and are not currently viable options at our institution ^12^.

Day of surgery cancellations affected 14.3% of all scheduled cases during our audit period. Whilst this number is not dissimilar to Argo *et al’s* analysis of urological cancellations in the Veterans Health Administration (14%), it still represents a significant amount of wasted ST ^11^. Whilst some of these cancellations were unavoidable, such as staff illness, the majority of the 6 cancellations secondary to patient factors (P1, P6, P8, P10) could potentially have been avoided by a phone call to the patient a day or two prior to the operative date, allowing adequate time for replacement cases to be found. The 11 cancellations due to workup and administration factors (W1, W2, W4B, W5, F6) could foreseeably have been avoided by better communication between members of the surgical, anaesthetic and nursing teams. If foreseen early on the day of surgery, these otherwise wasted operative minutes could have been filled with elective patients called in at short notice. Other units have established a “fillbuster” list for just such events ^9^. Whilst there was a significant proportion of our operative time wasted with day of surgery cancellations, we do acknowledge that implementing such a waitlist for elective surgery patients is logistically challenging in a smaller hospital such as ours, and is impractical for many of our patients.

During the audit, CT consumed 6.9% of all available sessional time, and whilst the mean CT was acceptable at 8 minutes, on 31 occasions a significant delay of over 15 minutes occurred. Harders *et al.* successfully reduced their turnover time by 37% by supplying pagers for all theatre assistants which were triggered 5 minutes prior to completion of the case in addition to standardisation of equipment ^2^. Whilst not an issue in our centre during the course of the audit, Weinbroum *et al’s* audit has shown that up to 10% of all available operating time is wasted awaiting Post-Anaesthetic Care Unit (PACU) space ^13^. Wasted operative time costs an estimated $US 10–30 per minute or at least $US 600 per hour ^13– 15^. As the hourly cost of maintaining a single patient in PACU costs $US 110, increased throughput and profitability could both be achieved by increasing the staffing and by physical enlargement of the PACU ^13, 16– 18^.

The greatest gains in terms of surgical throughput efficiency have been seen with implementation of parallel processing. Parallel processing involves preparing patients for theatre concurrently as the prior patient’s procedure is completed, contrasting to the traditional approach of serially processing patients. This approach allows for reduction of both AT and CT. Parallel processing allows intravenous and arterial lines to be inserted, and spinal or even general anaesthesia to be achieved in pre-procedure rooms ^5, 19^. Simultaneous processing often requires additional anaesthetic staffing and the consequent increased costs associated. However, the increased cost of implementing parallel processing can be offset by increased throughput and consequent financial gain ^6^.

Parallel processing is most advantageous in operating lists where multiple, small cases are going to be performed, in cases with a consistent operative duration, and on full day operating lists ^20^. These incremental time savings over preceding cases enable the performance of additional cases ^19, 21– 25^. Whilst parallel processing only facilitates added cases on high turnover lists, it has been shown to reduce overtime costings in theatres where fewer, longer cases are performed, however, in this circumstance this might not offset the added costs of increased staffing levels ^22, 26^. Sandberg *et al.* have shown that parallel processing, when used across an entire theatre complex, is cost neutral ^23^. More selective use of parallel processing, such as solely for high turnover lists, would yield the greatest benefit financially without affecting case throughput ^23^.

## Conclusion

Our study quantified how much of each theatre session was occupied by non-operative processes in a unit with multiple short-duration operative procedures. Based on the findings of the study, we have implemented process changes to increase our own theatre efficiency. We have recently adopted a high-throughput theatre list one day a month, where multiple small simple cases are booked on an all-day operating list. Additional anaesthetic and nursing staff are rostered on these days to facilitate parallel processing of patients and remove the need for lunch breaks. If these prove to be successful and cost-efficient we would look to increase their frequency.

Whilst we are unable to implement more complex booking algorithms at this stage due to financial constraints, we are still striving to improve our booking efficiency. Efforts are being made to more accurately document predicted operative times on booking forms. Uncomplicated patients are now having blood and urine taken when booked at outpatients. They are then being seen immediately by pre-operative nurses. This enables suitable patients to be prepared for surgery at short notice in the event of cancellations. Booking staff are now making efforts to contact patients several days prior to their surgery and in the event of problems, to liaise with the surgical team, to limit the number of day of surgery cancellations. Assessment of our unit’s theatre throughput is ongoing.

## Data availability

*F1000Research:* Dataset 1. Raw data files (Excel) for the audit of elective theatre operating in the Urology unit, Ipswich General Hospital, QLD, Australia, 10.5256/f1000research.4824.d32766 ^27^
