# Supplementary material for: Causes of elective surgery cancellation and theatre throughput efficiency in an Australian urology unit
Source: F1000Res. 2014 Aug 19;3:197. [Version 1] doi: 10.12688/f1000research.4824.1 (PMC4184311; doi:10.12688/f1000research.4824.1)
Supplement: Peer review history

# Peer review: Referee response for version 1

**DOI:** 10.5256/f1000research.5149.r6092

**Published:** 2014-09-10

**Authors:** M Hammad Ather

**Affiliations:** 1 Department of Surgery, Aga Khan University, Karachi, 74800, Pakistan

Operating room space and time is one of the major reasons for long waiting times in elective operative procedures. Improving operating room efficiency is therefore imperative. The authors of the current report attempted to identify lacunae in the operating room throughput and identify reasons for cancellations of elective surgeries.

The authors noted that a significant proportion of time spent is non operative. This is particularly important for urology, where a typical operating list is comprised of many short endoscopic procedures. The authors recommend increasing nursing and anesthetic staff to cater to this particular need. Induction rooms are valuable as they limit the loss of non operative utilization of the OR room. It is not apparent from current rooms whether the induction room was fully utilized in the course of this work.

Last minute cancellation of an operative procedure is not only an emotional and financial stress on the family/insurance but also on the hospital. Pre-operative anesthetic and nursing review is valuable in significantly reducing cancellations on the day of surgery.

I have read this submission. I believe that I have an appropriate level of expertise to confirm that it is of an acceptable scientific standard.

# Peer review: Referee response for version 1

**DOI:** 10.5256/f1000research.5149.r5860

**Published:** 2014-08-28

**Authors:** Levent Turkeri

**Affiliations:** 1 Department of Urology, Marmara University School of Medicine, Istanbul, Turkey

This is a prospective study over a 6 month period to identify the causative factors for delays or cancellations including time of patient arrival in anaesthetic bay, start time of operative procedure, end time of operative procedure, and time of patient leaving theatre.

In terms of non-operative time, anaesthetic process comprised the largest part with 8974 minutes (33.4%). Changeover time was approximately 7%. The most common reason for cancellation was lack of surgical fitness, followed by inadequate operative time.

This study sheds light for possible reasons of inefficient OR time usage in a certain institution which subsequently led to a change in their logistic planning. Similar studies must be repeated in different institutions in various countries in order to find out the common obstacles as well as possible particular local solutions that speeds up the process.

I have read this submission. I believe that I have an appropriate level of expertise to confirm that it is of an acceptable scientific standard.
